# Land grabbing: a preliminary quantification of economic impacts on rural livelihoods

**DOI:** 10.1007/s11111-014-0215-2

**Published:** 2014-07-08

**Authors:** Kyle F. Davis, Paolo D’Odorico, Maria Cristina Rulli

**Affiliations:** 1 Department of Environmental Sciences, University of Virginia, Charlottesville, VA 22904 USA; 2 Department of Civil and Environmental Engineering, Politecnico di Milano, 20133 Milan, Italy

**Keywords:** Land rush, Rural livelihoods, Small-holder agriculture, Developing world, Land access

## Abstract

Global demands on agricultural land are increasing due to population growth, dietary changes and the use of biofuels. Their effect on food security is to reduce humans’ ability to cope with the uncertainties of global climate change. In light of the 2008 food crisis, to secure reliable future access to sufficient agricultural land, many nations and corporations have begun purchasing large tracts of land in the global South, a phenomenon deemed “land grabbing” by popular media. Because land investors frequently export crops without providing adequate employment, this represents an effective income loss for local communities. We study 28 countries targeted by large-scale land acquisitions [comprising 87 % of reported cases and 27 million hectares (ha)] and estimate the effects of such investments on local communities’ incomes. We find that this phenomenon can potentially affect the incomes of ~12 million people globally with implications for food security, poverty levels and urbanization. While it is important to note that our study incorporates a number of assumptions and limitations, it provides a much needed initial quantification of the economic impacts of large-scale land acquisitions on rural livelihoods.

**Electronic supplementary material:**

The online version of this article (doi:10.1007/s11111-014-0215-2) contains supplementary material, which is available to authorized users.

## Introduction/Background

Population growth, dietary changes and increasing use of crop-based biofuel are placing ever greater demand on food production and its requisite resources (Godfray et al. 2010). In addition, climate change is projected to adversely affect reliable and sufficient food supply in the future (Cline 2007). These changes in the demand and supply of agricultural products threaten food and water security as well as sustainable livelihoods. Due to these demographic and environmental pressures and the 2008 food crisis, many nations and corporations with the requisite capital are making large-scale investments in agricultural lands both domestically and abroad to either accumulate a reliable reservoir of land and water resources in the event of increased climatic uncertainty or to speculate on the price of cultivatable lands (Deininger and Byerlee 2011). While the potential benefits (e.g., insurance against food price shocks, increased global food supply) of these deals may be apparent, such transactions often take place at the expense of and without informed consent from the prior land users (Anseeuw et al. 2012; Deininger and Byerlee 2011; Cotula et al. 2009). This fact has been the source of wide discussion in the land rush literature (e.g., Vermeulen and Cotula 2010; Zoomers 2010; Borras et al. 2011; De Schutter 2011), but is all too often overlooked by the involved governments and investors. These large-scale land acquisition projects often emphasize the rapid increase in yield that they can produce and the additional employment they can provide. However, the benefits of this additional agricultural production are often not felt locally (Anseeuw et al. 2012; D’Odorico and Rulli 2013), so that the loss of access to land can ultimately spell significant dietary, social, cultural and economic consequences for rural communities in the targeted areas (Borras et al. 2011; De Schutter 2011). Given the lack of transparency in many of these transactions, it is understandable that a quantitative literature on the human impacts of this phenomenon is sparse. Despite this apparent difficulty, several studies have been able to broadly assess the amount of land appropriated (e.g., von Braun and Meinzen-Dick 2009; Deininger and Byerlee 2011). However, knowing the area controlled by investors can only inform the discussion so much, and a more pointed quantification of the specific impacts of the global land rush is now necessary. That is why steps are now being taken in the land rush literature to turn the focus from studies purely assessing the area affected by such land deals toward quantification of the potential environmental and human impacts (Edelman 2013; Rulli et al. 2013; Rulli and D’Odorico 2013). One such study sought to quantify the potential for these land deals to impact malnourishment in the affected areas, estimating 200–300 million people at risk of greater food insecurity as a direct result (Rulli and D’Odorico 2014). Though this reduced ability to feed people locally is important to consider, it is only one way in which rural communities may experience the impacts of this global land rush. We focus here on a single question, namely: how many people in rural communities of targeted areas may potentially experience income loss as a direct impact of these agricultural land deals? We argue that, since the communities in these areas rely on agriculture for income, the loss of access to land and water resources as a result of land deals represents an inability to produce household income. While we only quantify this potential impact in lands intended for food crops, we should also note that large-scale land acquisitions can occur for several other reasons. For instance, recent increases in demand for agricultural land for biofuels is an effect of new energy policies (European Parliament 2009; UN DESA 2011) aimed at curbing the increase in atmospheric CO2 concentrations. Further, some large-scale investments in forested land can be driven by prospects of profitable investments in the carbon credit market for climate change mitigation (Meyfroidt et al. 2013; Fairhead et al. 2012; The Oakland Institute 2011c).

**Fig. 1:**
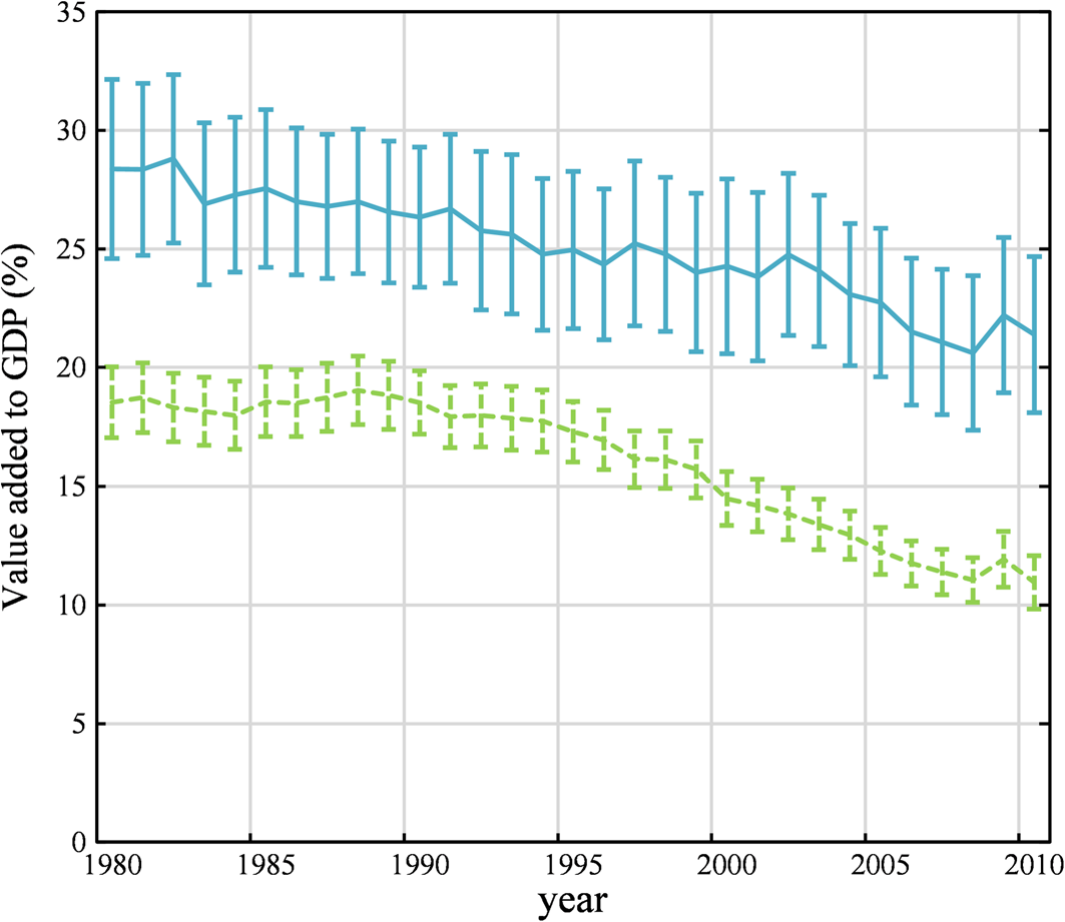
Average percent contribution by agricultural sector to gross domestic product for the 28 significantly grabbed countries (*solid*/*blue*) and all other countries (*dashed*/*green*) from 1980 through 2010. Error bars represent the standard error of the mean

Targeted countries typically have lower levels of development and economies heavily reliant on the agricultural sector, in terms of both employment and value of domestic product (Fig. 1), making the livelihoods of their citizenry especially sensitive to climatic change, land degradation and this recent global land rush (IFPRI 2012). Specifically here, we consider how targeted land resources that would otherwise be used for local crop production translate into a reduced ability to sustain the livelihoods of the current population in the affected areas. This is especially important considering that rural households in agriculture-based economies are limited in their opportunities for non-farm employment unrelated to agricultural production (FAO 2010a, b; UN DESA 2011). The income lost from targeted agricultural land represents a reduced ability of the area to support a certain number of people. Thus, while there may be various contributing factors to the problem, the sole impact we explore here is the income loss by rural communities as a result of large-scale land acquisitions and how this impact varies across the most targeted countries. By calculating the total lost income due to confirmed large-scale land deals, we examine the portion of a country’s population with the *potential* to be directly economically impacted by these land deals and briefly suggest (while citing the limited available evidence) that this may result in increased urbanization and human migrations in order for rural communities to diversify their incomes (Tacoli 2011; The Government Office for Science 2011; UN DESA 2011). This study provides an *initial,* but much needed, quantification of the number of rural people whose livelihoods may be potentially impacted by large-scale land acquisitions. By providing empirical support, the intent of our work here is to act as a stepping stone for further studies with more definitive conclusions and to direct the attention of land deal research toward better quantifying the impacts of land deals on rural populations. Just as with tropical deforestation or urbanization, the issue of large-scale land acquisitions is a rapidly evolving phenomenon (Cotula et al. 2014). This is compounded by the fact that information on land deals and their rural economic impacts suffers from a lack of transparency (Anseeuw et al. 2012; Rulli et al. 2013). Yet despite the difficulty that these issues can present in staying current with the dynamics of the phenomenon, our study provides a novel alternative approach with the potential to fill an important knowledge gap in our growing understanding of large-scale land acquisitions and their many possible impacts.

## Methods/Data

We study the 28 countries (Table 1) most targeted by large-scale land acquisitions [comprising 87 % of reported cases and 27 million hectares (ha)]. We define large-scale land acquisitions as transactions that target agricultural areas and that entail the transfer of rights to use, control or ownership through sale, lease or concession to commercial farming. Based on current yield scenarios (Mueller et al. 2012), country-specific crop yields for the year 2000 (which reflect national average yields before the land deal) were multiplied by land areas under contract from the new (June 2013) Land Matrix database (Land Matrix 2013) to calculate the agricultural production for each edible crop. Recently, criticism has been raised toward quantitative studies on large-scale land acquisitions relying on previous data sets of large-scale land acquisitions (Scoones et al. 2013; Oya 2013; Edelman 2013). Part of the criticism was based on lack of on-ground verification of the acquisition and on the fact that a substantial number of announced deals fail in the course of the negotiation stage. The new Land Matrix data set (Anseeuw et al. 2013; Land Matrix 2013) improves upon these criticisms and specifies whether each deal is just intended or concluded and also reports the area under contract. It also indicates whether the land has already been put under production by the investors (Land Matrix 2013). Here, we consider *only* concluded deals, for which the contract area was specified (Table S1), regardless of whether the land is under production because we assume that at this stage previous land users have already been excluded from accessing the acquired land. These criteria ensure that land rights have legally changed hands and that the ability of rural communities (who typically rely on traditional land tenure systems; Vermeulen and Cotula 2010; Anseeuw et al. 2012; White et al. 2012; Wily 2012) to access that land has been affected. The fact that these deals deny rural communities further access to agricultural land is all that is necessary for their incomes to be impacted. We readily acknowledge (as do the authors of the Land Matrix database) that conclusions from this database must be arrived at with caution and make every effort to ensure that our estimates are conservative. Also, since data are not available for the crops previously grown on targeted lands, our estimates of production represent the *potential* amount of crops able to be grown on these lands at current yields had the land continued to be available to local communities. We assume that the intended crop types were grown on the land prior to the land deal. This is reasonable since most prior land use is by smallholder agriculture (Robertson and Pinstrup-Andersen 2010; Anseeuw et al. 2012). This assumption in turn can lead to inconsistencies in certain instances (for sugar cane in particular) between FAO estimates of production and our own. As stated before, this is likely because targeted land may not yet be actively cultivated, but it is no longer accessible by rural communities. Thus, even if a community intended in the coming years to expand cultivation onto land that is now incorporated, this community would no longer have that option. Gross agricultural production values (USD $ for crops used as food, feed or seed) and gross agricultural production (tons of crop production used as food, feed or seed) were obtained from the FAOSTAT database (FAO 2013). Unit prices of crops were calculated as the total gross agricultural production value of each crop by country divided by the gross agricultural production of that crop for that country (FAO 2013). A value of $484 per metric ton was used for missing oil palm data, as this was the unit value given by the FAO for all African countries considered where data were available. To account for production costs, we first took the sum of the gross capital stock for the year 2007 (the most recent year available) for land development, plantation crops, machinery and equipment (FAO 2013). We then divided this by the total gross production value of crops by country to obtain the national average fraction of gross agricultural value lost to production costs. This further ensures that our estimate is conservative since a portion of the gross capital stock considered also takes into account land development, machinery and equipment used for livestock production and thus is an overestimate. We do not consider the cost of fertilizers, as sub-Saharan Africa, Latin America and South-East Asia have low levels of synthetic fertilizer consumption (Morris et al. 2007; Potter et al. 2010). We also do not consider transportation since the gross agriculture value represents the value of the production at farm gate. Oil palm production was converted to palm oil production by country-specific ratios of palm oil production to oil palm fruit production obtained from FAOSTAT (FAO 2013). The appropriate unit price was multiplied by the quantity of lost agricultural production, and the sum of these crop values gave the total lost agricultural income by a country as a result of recent land deals. This total was then divided by the average income per capita (The World Bank 2013) to give the number of people who could *potentially* lose their income as a result of large-scale land acquisitions (see supplementary materials for more details). Since data on average rural income were not available for the countries of interest, average income per capita was given as the gross national income (GNI) per capita in terms of purchasing power parity. These data were from the World Bank’s World Development Indicators database (The World Bank 2013), as were population data for each country and percent value added by the agricultural sector. The use of GNI (as opposed to rural per capita income) may, in turn, underestimate the total number of people affected, thus ensuring that our estimate errs on the conservative side. Jatropha was conservatively excluded from these calculations because: (1) it is not yet clear if the crop is profitable and (2) it is typically grown on marginal land (Brown 2012). Data for the percent value added by agriculture to GDP were from the World Bank’s World Development Indicators database (The World Bank 2013).

**Table 1:** Summary findings for grabbed countries

	Total lost income ($)	Total people affected	% of population
Angola	79,337,812	15,383	0.08
Argentina	345,949,205	22,342	0.06
Benin	16,783,119	10,614	0.12
Brazil	454,969,840	41,386	0.02
Cameroon	203,675,121	90,845	0.46
Colombia	403,308,909	44,722	0.10
Congo	13,127,064	4,136	0.10
DRC	105,572,483	319,605	0.48
Ethiopia	809,980,299	785,701	0.95
Gabon	1,440,146,140	110,167	7.32
Ghana	332,672,327	206,456	0.85
Guatemala	68,573,647	14,817	0.10
Indonesia	7,736,024,665	1,847,609	0.77
Liberia	225,161,293	478,476	11.98
Madagascar	158,298,340	165,997	0.80
Malaysia	8,956,266,573	608,958	2.14
Morocco	926,336,692	201,836	0.63
Mozambique	2,443,013,473	2,710,813	11.59
Nigeria	331,781,421	153,439	0.10
Papua New Guinea	3,758,184,784	1,564,440	22.81
Peru	119,124,632	13,524	0.05
Philippines	804,018,409	203,256	0.22
Russia	27,585,683	1,423	<0.01
Sierra Leone	501,467,190	610,031	10.40
South Sudan & Sudan	3,561,260,372	1,731,108	3.97
Tanzania	305,055,452	215,955	0.48
Uganda	19,237,881	15,379	0.05
Uruguay	115,090,195	8,483	0.25
Total	34,262,003,020	12,196,904	–

As Edelman (2013) states, studies of insufficient rigor (both in the quality of data/analyses used and the unjustified conclusions drawn) can be counterproductive and act to hurt the case of rural communities, as such studies will not hold up to scrutiny by opposing viewpoints. Our work here makes every effort not to do so by: (1) using an up-to-date database (that has addressed much of the criticism of its preceding versions; Anseeuw et al. 2013) and rigorous criteria to select land deals to include in our analysis, (2) using a simple, conservative yet powerful analysis to estimate the impact on rural income and (3) seeking only to approximate the *potential* number of rural people affected by large-scale land acquisitions [making no claims at establishing a conclusive “killer fact” as several authors caution against (Oya 2013; Scoones et al. 2013)]. Lastly, we should note that while the database used in this study is a significant improvement on its previous versions, it is still subjected to certain biases (e.g., countries’ data policies, focus on international investments), which should be taken into consideration when drawing any conclusions.

## Results

**Fig. 2:**
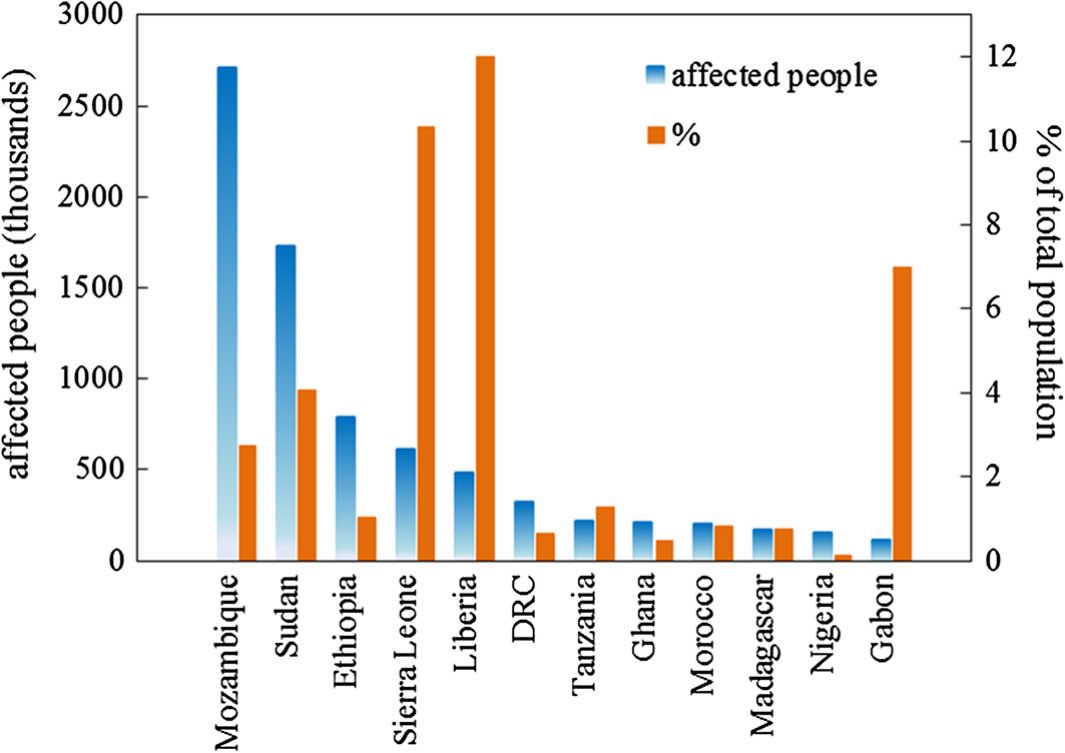
Summary for grabbed African countries. Chart shows African countries with more than 100,000 people potentially affected by land grabbing. Percent of total population is relative to the 2010 national populations

We estimate that in the 28 countries most affected by land deals from the year 2000 to present, more than 12.1 million people are potentially affected by the direct economic consequences of land acquisitions (Table 1). The percent of a population potentially affected by lost income due to this phenomenon falls below 1 % for all but 7 countries (Gabon, Liberia, Malaysia, Mozambique, Papua New Guinea, Sierra Leone and South Sudan/Sudan). However, the impact on lost livelihood varies widely by country. In Papua New Guinea for example, an income that could support nearly one quarter (23 %) of the population is potentially lost. Conversely, in countries such as Russia (<0.01 %), Brazil (0.02 %), Peru (0.05 %) and Uganda (0.05 %), the relative impact on employment prospects is minimal. Of the countries in this study, 16 have a potential lost income equating to greater than 100,000 people, and 4 have >1.5 million people potentially affected. In absolute numbers, Mozambique tops the list with more than 2.7 million people, followed by Indonesia (1.8 million), South Sudan/Sudan (1.7 million), Papua New Guinea (1.5 million) and Ethiopia (0.78 million). Since there are no data in the peer-reviewed literature supporting these findings (Robertson and Pinstrup-Andersen 2010), comparisons are limited. However, several reports from NGOs indicate that our estimates are reasonable. For instance, our estimate for Ethiopia agrees well with a report (The Oakland Institute 2011b) placing the number of affected people at 1 million. Our approximation for Uganda corresponds well to an estimate for select affected districts of 20,000 people (Oxfam International 2011). Also, a major land deal in Tanzania will reportedly displace more than 160,000 people (The Oakland Institute 2011a). According to our findings, the regions with the potential to be most heavily impacted in terms of lost agricultural income are sub-Saharan Africa and Southeast Asia. While Africa accounts for 43 % of the appropriated area in this study, Africans comprise roughly two-thirds (8.2 million people) of all those potentially affected (Fig. 2). We estimate total lost income globally at ~$34 billion, a number comparable to the ~$35 billion loaned by the World Bank for development and aid in 2012 (The World Bank 2012). The local agricultural livelihoods of smaller countries in West Africa appear to be particularly vulnerable to the potential effects of land acquisition (Fig. 2). Again, we stress that the results presented here are conservative estimates. The analysis here thus provides a new and simple way to quantify a phenomenon with a reputation for lack of transparency and to gain a first approximation of how severely impacts on rural income may be across countries.

## Discussion

From the outset of this discussion, it is important to note that while this study contributes important empirical evidence of the economic impacts of the global land rush on rural communities, the findings should by no means be viewed as conclusive. They should instead be considered as an upper limit (of potential impacts on rural people) against which future case studies can be measured. This is particularly noteworthy given the significant assumptions incorporated into our methodology (especially related to prior use and crop type) and, in turn, how they may influence our findings. Despite this, we find that where data are available our results agree quite well with case studies where rural communities were either displaced or their livelihoods were affected.

While the loss of income and employment opportunities by rural communities is an important impact to consider, we also acknowledge that with each land deal comes a unique set of benefits to, impacts on and responses by the affected local communities (Borras and Franco 2013). The fact of varying benefits, impacts and responses is true both between and within countries, as was highlighted in McCarthy’s work (2010) in Indonesia. Here, he noted that the options presented to smallholders and the ways in which they choose to interact with commercial agriculture ultimately dictate whether change is positive or negative. In addition, as Borras and Franco (2012) have previously described, the perspective from which a land deal is viewed plays an important role in how benefits of land deals are defined and whether they have been realized. For instance, a land deal that improves crop production or rural employment opportunities may result in environmental degradation. While potential benefits and impacts vary with each case and for each stakeholder, Li’s work (2011) examining existing data on the land rush phenomenon and taken from a labor perspective demonstrates that poverty reduction is an unlikely result of large-scale land acquisitions. However, the question of benefits is far more certain at the national level for the target country where land deals are more likely to result in some economic and political benefits (Li 2011). This was notably the case in a study by the organization Welthungerhilfe of a recent land deal in Sierra Leone (IFPRI 2012; Melbach 2012). In this instance, local farmers were denied access to land without prior consultation and experienced a drastic loss of reliable income, making them less able to afford food for their households and school fees for their children. Except for a small one-time payment to farmers of USD 220 and minimal annual area-based payments of USD 6.25 per hectare for oil palm land only (compared to an average annual GNI per capita of USD 880), farmers are unable to obtain income from the land. Conversely, the various levels of government administration receive the other 50 % of the investors’ yearly lease payment.

While this is a compelling example of what we seek to examine here, what is more broadly essential to consider is to what extent the potential benefits from land contracts (and the activities that follow) actually find their way to the populace just as the original agricultural income would. One way by which these changes in land tenure can potentially benefit and sustain the livelihoods of local communities is by providing employment opportunities with adequate income. While investing corporations regularly make estimates on new job creation, the actual number of jobs created is typically well below expectations, due to transitions to plantation style agriculture preferring mechanization and wage laborers (Deininger and Byerlee 2011; Cotula et al. 2009). In most cases, the opportunities for employment are low-quality and limited or nonexistence (Deininger and Byerlee 2011; Cotula et al. 2009; Li 2011). Moreover, land acquisitions largely affect rural (and generally poorer) communities in countries where wealth tends to be distributed less equally. Overall this means that vulnerable communities within vulnerable countries (i.e., those most impacted by changes in food prices) are also those more susceptible to livelihood loss due to the land rush. Where agricultural production is primarily contributed by subsistence farming, the loss of cropland can also be interpreted as a reduced ability to meet the dietary requirements of a targeted country’s population (The Oakland Institute 2011b; Rulli and D’Odorico 2014).

The extent to which these land deals potentially affects employment prospects within a country varies widely and is unique to each case. The number of people potentially affected ranges from thousands to millions (1), highlighting the fact that countries are differentially affected by and sensitive to consequences of large-scale land acquisitions. As per capita income can vary greatly between targeted countries (e.g., USD 330 per year in the Democratic Republic of the Congo vs. USD 14,680 per year in Malaysia; World Bank 2013), a person’s average income is an important consideration in assessing the consequences of such land deals. While we make every effort to keep our estimates conservative, our approximations of the potential number of people affected by income loss due to the global land rush provide important insight into which countries may expect to experience this impact most heavily (even if the country’s land area under contract is comparatively small; e.g., Mozambique). How much of a country’s income comes from agriculture (Fig. 1) and how many of its people are employed in that sector (Table 1) contribute to how vulnerable a country may be to the effects of large-scale land acquisitions. The strength of a targeted country’s legal system, the extent of enforcement and the ease for investing countries in navigating its land tenure system also help determine which places are preferentially targeted (Anseeuw et al. 2012; Deininger and Byerlee 2011; Rulli et al. 2013). Ultimately, this can lead to the sudden marginalization of rural communities and leave them with limited options for alternative forms of household income. To worsen this vulnerability, less-developed countries (and rural areas in particular) are predicted to experience a disproportionately large amount of the adverse consequences of climate change (Rosenzweig and Parry 1994; Parry et al. 2004). While the analysis here focuses on people, the consequences of environmental change are likely to be compounded with a transition to a more commercialized means of agricultural production. These adverse effects typically associated with transition to commercial-scale agriculture include pollution from increased fertilizer usage and soil loss from mechanized planting and harvesting (Brown 2012). However, since the land rush has only taken place in the past several years, many of these potential adverse effects may require more time to be fully discernible. This is true not only for environmental impacts, but as Cotula et al. (2014) point out, also for impacts on rural livelihoods, since land deals across the world are at various stages of establishment and implementation. The fact that many land deals have taken longer than expected to implement can also mean significant opportunity costs, where it becomes less likely that positive outcomes will counter negative impacts (Cotula et al. 2014).

From the perspective of local communities, the economic consequences of land deals can often be thought of as analogous to those of crop failures. In both cases, the financial (e.g., transportation fare) and infrastructural (e.g., roads, bridges) means to seek employment through non-farm activities are often left intact, but the enduring livelihoods of households are threatened. Given the proximity of many land deals to urban areas (Anseeuw et al. 2012), the prospect of migration becomes all the more reasonable. In Bangladesh, a place visibly experiencing the early effects of climate change through increased flooding, it was found that crop failures (and not flooding) better explained people’s propensity to migrate permanently (Gray and Mueller 2010). Thus, as with land acquisitions, loss of local profit from crop production for the foreseeable future can make migration a reasonable option for securing a household’s income (Tacoli 2011; The Government Office for Science 2011). Similarly in China, migration due to the conversion from subsistence farming to commercial agriculture has been reported (Siciliano 2013). Also, in Ethiopia, large-scale land acquisitions have reportedly caused transboundary displacements of local farmers and pastoralists into Sudan (The Oakland Institute 2011b).

## Conclusion

Overall, how affected communities are able to financially cope with the impacts of these land deals depends upon their access to assets, infrastructure and opportunities (UN DESA 2011).The effects of large-scale land investments can be multitudinous, with advocates on either side touting their positives (e.g., technology sharing, increased crop yields) and negatives (e.g., lost livelihoods, unjust land appropriations, environmental degradation) (D’Odorico and Rulli 2013; Toft 2013). This study offers a first insight into the impact of the recent land rush on rural livelihoods. Our conservative estimate of over 12 million people losing their incomes is more than one-third of the number of internally displaced people due to conflict (29 million people) (IDMC 2013a) and one quarter of the number of migrations induced by natural hazards in 2012 (32 millions) (IDMC 2013b). This relatively large number of people may contribute to issues of food insecurity and poverty in rural areas while challenging the sustainability of urban growth as affected people seek to diversify household income (UN DESA 2011).

Losing access to land can carry with it a variety of economic, social, nutritional and cultural consequences (De Schutter 2011), a full discussion of which is beyond the scope of this study. Income loss represents just one way through which these deals might adversely affect rural communities. By quantifying the number of rural people potentially affected by these land deals, we can also begin to understand the extent of the social and cultural impacts, an equally important aspect of the on-going conversation surrounding the global land rush (Edelman 2013). Our study provides estimates from an economic perspective against which field studies can be compared. Given the lack of transparency of this phenomenon, these findings provide a much needed *initial* empirical evaluation of the direct impacts of large-scale land acquisitions on rural communities and their livelihoods. While our study and others like it (Rulli and D’Odorico 2013) are a good first step, on-ground verification is an essential next step toward firmly quantifying the human impacts of this process (Scoones et al. 2013; Oya 2013; Edelman 2013). Where our estimates best agree with such verifications can provide valuable information as to the primary impact (i.e. income loss) on rural households (and its magnitude) in these areas and help direct possible ways to address the problem.

## Electronic supplementary material

Below is the link to the electronic supplementary material.

Supplementary material 1 (DOC 141 kb)
