# Supplementary material for: Land grabbing: a preliminary quantification of economic impacts on rural livelihoods
Source: Popul Environ. 2014 Jul 8;36(2):180–92. doi: 10.1007/s11111-014-0215-2 (PMC4223572; doi:10.1007/s11111-014-0215-2)
Supplement: Supplementary file 1 — Supplementary material 1 (DOC 141 kb) [file 11111_2014_215_MOESM1_ESM.doc]

Supplementary Materials

Tables:

Table S1. List of countries and specific crops. For each crop, the grabbed area, yield, net unit price (given as the rounded whole number of the value used) and gross production value (and conversion factor for oil palm) are given. The yield value for bananas grown in Cameroon was used for bananas in Nigeria. The average African oil palm conversion efficiency and unit price were used for Ethiopia, South Sudan/Sudan and Uganda. The unit price for citrus fruit in Morocco was calculated as the production-weighted average of clementines, lemons, limes, oranges, mandarins and tangerines.

| **Country** | **Crop** | **Area (ha)** | **Yield (t/ha)** | **Unit Price (USD/t)** | **Conversion factor** | **Gross production value lost (USD)** |
| --- | --- | --- | --- | --- | --- | --- |
| **Angola** | Oil Palm | 81,500 | 4.7 | 484 | 0.196 | 36,332,951 |
|  | Rice | 101,500 | 1.4 | 309 |  | 43,004,861 |
| **Argentina** | Maize | 40,331 | 5.6 | 157 |  | 35,703,396 |
|  | Rapeseed | 146,998 | 1.3 | 310 |  | 58,709,970 |
|  | Rice | 57,331 | 5.3 | 310 |  | 94,537,539 |
|  | Sorghum | 42,650 | 4.6 | 171 |  | 33,710,629 |
|  | Soybeans | 156,501 | 2.2 | 304 |  | 105,774,351 |
|  | Wheat | 42,555 | 2.4 | 175 |  | 17,513,319 |
| **Benin** | Cassava | 1,000 | 11.0 | 111 |  | 1,222,950 |
|  | Maize | 1,667 | 1.1 | 141 |  | 267,083 |
|  | Oil Palm | 1,667 | 10.8 | 484 | 0.169 | 1,469,594 |
|  | Rice | 16,000 | 2.0 | 231 |  | 7,406,623 |
|  | Soybeans | 1,667 | 0.5 | 304 |  | 238,518 |
|  | Sugar Cane | 4,800 | 35.3 | 36 |  | 6,178,350 |
| **Brazil** | Maize | 21,500 | 2.9 | 157 |  | 9,939,023 |
|  | Oil Palm | 59,550 | 8.7 | 482 | 0.214 | 53,740,609 |
|  | Soybeans | 21,500 | 2.4 | 304 |  | 15,709,482 |
|  | Sugar Cane | 148,550 | 69.4 | 36 |  | 375,580,725 |
| **Cameroon** | Bananas | 24,930 | 7.3 | 313 |  | 56,575,778 |
|  | Cassava | 12,465 | 11.5 | 116 |  | 16,663,818 |
|  | Maize | 12,465 | 2.0 | 157 |  | 3,959,105 |
|  | Oil Palm | 71,504 | 15.5 | 630 | 0.149 | 104,109,260 |
|  | Rice | 12,465 | 3.2 | 309 |  | 12,298,471 |
|  | Sugar Cane | 29,141 | 9.5 | 36 |  | 10,068,689 |
| **Colombia** | Oil palm | 157,876 | 11.9 | 482 | 0.251 | 227,863,360 |
|  | Sugar Cane | 125,000 | 38.5 | 36 |  | 175,445,549 |
| **Congo** | Cassava | 1,000 | 9.0 | 116 |  | 1,043,522 |
|  | Maize | 1,667 | 0.8 | 157 |  | 209,583 |
|  | Oil Palm | 1,667 | 12.4 | 483 | 0.18 | 1,794,875 |
|  | Rice | 16,000 | 0.7 | 309 |  | 3,515,182 |
|  | Sugar Cane | 4,800 | 37.5 | 36 |  | 6,563,902 |
| **DRC** | Maize | 1,016 | 0.8 | 157 |  | 127,623 |
|  | Oil Palm | 388,180 | 3.4 | 483 | 0.161 | 102,531,227 |
|  | Rice | 7,046 | 0.7 | 309 |  | 1,612,736 |
|  | Soybeans | 8,062 | 0.5 | 304 |  | 1,300,896 |
| **Ethiopia** | Maize | 134,994 | 1.9 | 157 |  | 39,905,399 |
|  | Oil Palm | 238,326 | 3.3 | 483 | 0.171 | 64,565,033 |
|  | Rice | 257,482 | 0.7 | 309 |  | 51,764,249 |
|  | Soybeans | 130,721 | 2.1 | 297 |  | 81,799,019 |
|  | Sugar Cane | 186,496 | 80.1 | 36 |  | 544,318,670 |
|  | Wheat | 132,571 | 1.2 | 175 |  | 27,627,929 |
| **Gabon** | Oil Palm | 1,325,016 | 7.9 | 483 | 0.2 | 1,013,487,025 |
|  | Sugar Cane | 226,952 | 51.6 | 36 |  | 426,659,115 |
| **Ghana** | Bananas | 17,570 | 2.9 | 339 |  | 17,047,018 |
|  | Cassava | 3,000 | 11.7 | 116 |  | 4,062,770 |
|  | Maize | 162,774 | 1.5 | 157 |  | 37,111,676 |
|  | Oil Palm | 201,945 | 9.1 | 481 | 0.062 | 54,660,382 |
|  | Rice | 171,580 | 1.9 | 302 |  | 98,602,107 |
|  | Sugar Cane | 133,333 | 24.9 | 36 |  | 121,188,373 |
| **Guatemala** | Oil palm | 66,718 | 12.7 | 483 | 0.15 | 61,324,724 |
|  | Sugar Cane | 2,500 | 79.2 | 37 |  | 7,248,923 |
| **Indonesia** | Oil Palm | 3,597,597 | 16.4 | 482 | 0.215 | 6,102,248,153 |
|  | Sugar Cane | 826,293 | 54.3 | 36 |  | 1,633,776,512 |
| **Liberia** | Oil Palm | 588,948 | 3.3 | 484 | 0.241 | 225,161,293 |
| **Madagascar** | Maize | 26,667 | 0.9 | 157 |  | 3,731,800 |
|  | Oil Palm | 9,100 | 8.6 | 484 | 0.191 | 7,195,111 |
|  | Potatoes | 150 | 5.8 | 187 |  | 161,858 |
|  | Rice | 10,000 | 2.0 | 309 |  | 6,154,925 |
|  | Sugar Cane | 130,000 | 28.8 | 38 |  | 141,054,645 |
| **Malaysia** | Oil palm | 5,192,468 | 17.9 | 483 | 0.2 | 8,956,266,573 |
| **Morocco** | Citrus | 350,000 | 9.2 | 216 |  | 692,594,280 |
|  | Olives | 350,000 | 0.7 | 902 |  | 233,742,412 |
| **Mozambique** | Bananas | 18,800 | 6.4 | 872 |  | 105,357,132 |
|  | Maize | 27,134 | 0.9 | 132 |  | 3,287,705 |
|  | Pineapples | 174,552 | 6.8 | 1821 |  | 2,174,558,887 |
|  | Rice | 31,800 | 1.1 | 443 |  | 15,760,238 |
|  | Sugar Cane | 279,393 | 14.6 | 35 |  | 144,049,511 |
| **Nigeria** | Cassava | 198,264 | 10.1 | 116 |  | 232,178,816 |
|  | Maize | 6,335 | 1.1 | 157 |  | 1,115,695 |
|  | Oil Palm | 26,787 | 2.6 | 348 | 0.162 | 3,896,213 |
|  | Rice | 224,183 | 1.3 | 309 |  | 92,219,983 |
|  | Sorghum | 7,000 | 1.1 | 171 |  | 1,338,380 |
|  | Soybeans | 4,085 | 0.8 | 304 |  | 1,032,334 |
| **Papua New Guinea** | Oil Palm | 2,140,539 | 11.0 | 483 | 0.276 | 3,130,086,466 |
|  | Sugar cane | 315,005 | 54.7 | 36 |  | 628,098,318 |
| **Peru** | Oil Palm | 16,834 | 2.8 | 845 | 0.138 | 5,434,684 |
|  | Sugar Cane | 104,831 | 29.2 | 37 |  | 113,689,948 |
| **Philippines** | Bananas | 32,266 | 12.9 | 313 |  | 130,010,558 |
|  | Cassava | 20,000 | 5.3 | 116 |  | 12,359,922 |
|  | Maize | 153,500 | 0.9 | 157 |  | 20,998,371 |
|  | Oil Palm | 70,000 | 12.1 | 484 | 0.174 | 71,321,717 |
|  | Pineapples | 21,833 | 36.3 | 316 |  | 250,743,882 |
|  | Rice | 253,671 | 2.8 | 309 |  | 219,684,572 |
|  | Sugar Cane | 50,000 | 65.9 | 30 |  | 98,899,386 |
| **Russia** | Sugar Beet | 30,000 | 19.3 | 48 |  | 27,585,683 |
| **Sierra Leone** | Cassava | 144,051 | 5.2 | 116 |  | 86,517,319 |
|  | Maize | 31,823 | 0.9 | 157 |  | 4,653,636 |
|  | Oil Palm | 701,057 | 3.3 | 483 | 0.239 | 265,634,518 |
|  | Rice | 146,727 | 1.1 | 309 |  | 49,465,898 |
|  | Sugar Cane | 37,085 | 70.4 | 36 |  | 95,195,819 |
| **South Sudan & Sudan** | Maize | 681,169 | 0.7 | 157 |  | 79,253,919 |
|  | Oil Palm | 438,101 | 3.3 | 483 | 0.171 | 118,686,153 |
|  | Rice | 113,346 | 1.5 | 309 |  | 51,536,481 |
|  | Sorghum | 534,622 | 0.6 | 171 |  | 53,847,119 |
|  | Sugar Cane | 667,078 | 78.4 | 36 |  | 1,906,371,006 |
|  | Sunflower | 213,323 | 0.7 | 70 |  | 10,941,060 |
|  | Wheat | 2,107,995 | 2.3 | 273 |  | 1,340,624,635 |
| **Tanzania** | Barley | 2,940 | 0.1 | 132 |  | 46,595 |
|  | Maize | 18,570 | 1.5 | 157 |  | 4,350,670 |
|  | Oil Palm | 55,118 | 5.3 | 485 | 0.098 | 13,757,985 |
|  | Rice | 40,932 | 1.4 | 309 |  | 18,230,367 |
|  | Sorghum | 70,000 | 1.9 | 171 |  | 23,182,712 |
|  | Sugar Cane | 74,000 | 91.0 | 36 |  | 245,487,123 |
| **Uganda** | Oil Palm | 71,012 | 3.3 | 483 | 0.171 | 19,237,881 |
| **Uruguay** | Barley | 71,345 | 0.9 | 132 |  | 8,102,809 |
|  | Maize | 71,345 | 3.5 | 157 |  | 38,702,819 |
|  | Soybeans | 71,345 | 1.9 | 304 |  | 41,921,226 |
|  | Wheat | 71,345 | 2.1 | 175 |  | 26,363,342 |
| **Total** |  | **27,107,998** |  |  |  | **34,262,003,020** |
